# Design of an endoscopic OCT probe based on piezoelectric tube with quartered outside electrodes

**DOI:** 10.3389/fbioe.2024.1391630

**Published:** 2024-04-24

**Authors:** Jinyuan Hu, Sujian Wu, Guohua Shi, Jinyu Fan, Haoyang Yu, Sixu Chen

**Affiliations:** ^1^ School of Biomedical Engineering (Suzhou), Division of Life Sciences and Medicine, University of Science and Technology of China, Hefei, China; ^2^ Jiangsu Province Key Laboratory of Medical Optics, Suzhou Institute of Biomedical Engineering and Technology Chinese Academy of Science, Suzhou, China

**Keywords:** endoscopic OCT probe, piezoelectric ceramic tube, optical coherence tomography (OCT), imaging in ophthalmology, single-mode fiber

## Abstract

**Introduction:** Optical coherence tomography (OCT) is a pivotal imaging modality in ophthalmology for real-time, *in vivo* visualization of retinal structures. To enhance the capability and safety of OCT, this study focuses on the development of a micro intraocular OCT probe. The demand for minimal invasiveness and precise imaging drives the need for advanced probe designs that can access tight and sensitive areas, such as the ocular sclera.

**Methods:** A novel OCT probe was engineered using a piezoelectric tube with quartered electrodes to drive Lissajous scanning movements at the end of a single-mode fiber. This design allows the probe to enter the eyeball through a scleral opening. Structural innovation enables the outer diameter of the endoscopic OCT probe to be adjusted from 13G (2.41 mm) to 25G (0.51 mm), accommodating various imaging field sizes and ensuring compatibility with different scleral incisions.

**Results:** The fabricated micro intraocular OCT probe successfully performed preliminary imaging experiments on *in vivo* fingers. The Lissajous scanning facilitated comprehensive coverage of the target area, enhancing the imaging capabilities.

**Discussion:** The integration of a piezoelectric tube with quartered outside electrodes into the OCT probe design proved effective for achieving precise control over scanning movements and adaptability to different surgical needs. The design characteristics and practical applications demonstrated the probe’s potential in clinical settings.

## 1 Introduction

Optical Coherence Tomography (OCT) is a non-invasive high-resolution tomography method that utilizes the low-coherence interference principle of light to image inside bio-logical tissue to be detected (Huang et al., 1991). The combination of the endoscopy head and OCT has a wide range of applications, such as fundus retinal imaging (Joos and Shen, 2013), vascular endoscopy imaging (Yu et al., 2017), gastrointestinal endoscopy imaging (Masci et al., 2009), and tooth root crack detection (Wei et al., 2019) and other clinical applications, which can realize non-invasive living tissue high-resolution tomography imaging of human internal organs.

Ophthalmic endoscopy OCT is a special kind of OCT that uses the endoscopic OCT probe mechanism to bypass some opaque medium and directly perform structural imaging. It can display the cross-sectional outline of the large transverse field of view on the retina while reducing the influence of cloudy fluid on the fundus tissue structure imaging so that the surgeon can obtain enough information during surgery to complete intraoperative decision-making. For example, the observation of separation during retinal detachment surgery. At the same time, with the rapid development of robots today, ophthalmic surgical robots have been able to complete clinical surgical trials such as retinal stem cell injection. Endoscopic OCT will effectively solve the problem that the field of vision is not enough during robot surgery, which can ensure the safety of clinical surgical trials.

At present, the fundus OCT imaging system usually uses external irradiation. The light beam is used to image the macula, optic nerve, retinal nerve fiber, and choroid of the fundus (posterior pole of the eye) through the optical system of the cornea, pupil, and crystalline lens (Joos and Shen, 2013), and the field of view is severely limited. The low resolution of images obtained by external OCT may also occur due to optical aberration of media in the eye (Kang et al., 2010). In addition, distortion or turbidity of subjects’ eye media, such as dense cataracts and other factors, will reduce externally transmitted OCT signals and reduce the signal-to-noise ratio of the fundus image (Kang et al., 2010). In addition, endoscopy OCT can display the cross-sectional outline of the upper retinal membrane, avoiding the forced use of potentially toxic dyes to visualize the membrane due to insufficient technology (Almony et al., 2012). The use of ophthalmic endoscopy OCT imaging can well solve the three problems mentioned above in the fundus OCT imaging system of external irradiation. At the same time, optical coherence tomography (OCT), a non-invasive high-resolution non-destructive tomography technology, is introduced into ophthalmic surgery to improve the success rate of ophthalmic diseases and avoid using contrast media. Reduce surgical complications.

In some robots capable of performing ophthalmic microsurgery, OCT integrates surgical instruments as an important auxiliary part of the surgical robot, which realizes cooperation with the operator to accurately achieve synchronous and relative movement. The probe enters the eye through the scleral opening with a maximum acceptable diameter of 3 mm (Hayashi et al., 2014) for endoscopy imaging, which is more conducive to real-time observation of surgery and surgical conditions, to guide tissues to avoid Angle, lens and other important ocular tissue structures, avoid secondary damage (Yu et al., 2015), and improve the safety and controllability of surgical robot operation.

Using the probe to enter the eyeball can properly solve the problem that the opaque tissue and turbidity media in the eye limit the imaging of the fundus retina (Bouma et al., 2000) and other structures. The external handheld probe has a very compact structure, which can be entered into the eyeball to perform OCT imaging on the fundus tissue structure after minimally invasive treatment of the eyeball, and the problem of mutual interference with the volume and position of optical devices such as optical microscopes is greatly optimized.

In 2005, N. V. Iftimia et al. (Iftimia et al., 2005) developed a handheld OCT A-scan imaging system with a central wavelength of 1310 nm to measure depth-resolved information in solid tissue. The designed probe diameter was 250 μm, but this design could not provide two-dimensional information unless the entire probe was moved to produce a scan.

In 2013, Joos et al. (Joos and Shen, 2013) proposed a smaller, independent 25G handheld forward imaging B-scanning OCT probe. The scanning probe uses a coil magnetic oscillator to drive the fiber for scanning, and the mechanism to generate the fiber tip scanning is formed by sliding a 28G straight thin-walled stainless-steel tube along the curved part of the 34G probe tube. When the 28G probe slides along the 34G probe, it forces the tip of the 34G probe to drive the 125 μm single-mode fiber to vibrate in the aerated space for lateral scanning to achieve endoscopic OCT imaging.

In 2016, Asami et al. (2016) proposed a 23G intraoperative handheld OCT probe with optical fiber scanning driven by motors. With this handheld OCT probe, researchers successfully achieved *in vitro* OCT imaging of pig eye and rabbit eye retinal tissues. Subsequently, it was successfully applied to human clinical cases to distinguish the retina, optic disc, choroid, and other fine intraocular structures through OCT imaging.

In 2016, Mura et al. (2016) performed an OCT endoscopy with a 23G side-scanning SD-OCT probe, confirming that the use of intraocular SD-OCT can expand visual cues during surgery, help the decision-making process, and improve the outcome of surgery. Intraoperative OCT images of 7 patients with vitreoretinal disease were obtained by 20G probes.

Endoscopic OCT probes can be divided into two types according to different scanning modes: side-imaging (Mura et al., 2016) and forward-imaging (Joos and Shen, 2013). Side-imaging means that the scan is emitted and received by the side of the probe, and forward-imaging means that the scan light is emitted and received by the front end of the probe. From the perspective of safety, applicability, image quality, and entrance incision, the forward mode is more suitable for ophthalmic endoscopic OCT imaging. At present, the driving methods of endoscopic OCT probes mainly include Micro-Electro-Mechanical Systems (MEMS) (Zhang et al., 2015), motors (Asami et al., 2016), electromagnetic driving (Joos and Shen, 2013), piezoelectric material driving, etc. MEMS and motors are generally suitable for lateral scanning, and their size limits the size reduction of the endoscope needle.

Due to the selection of driving mode, the current endoscopic OCT probes used for ophthalmic imaging are constrained by the reduction of probe size, and the scanning mode is constrained by lateral scanning, which is not conducive to the safety and improvement of imaging quality of ophthalmic endoscopy. In this paper, the driving mode of the four-part piezoelectric ceramic tube is selected, and the input signal control is more convenient. The dimensions of the PZT tube used in this study were 35 mm in length, 5.7 mm in outer diameter, and 0.67 mm in wall thickness. The size of the PZT tube does not limit the size reduction of the probe, which greatly reduces the fabrication cost of the probe. The biggest difference with our endoscopic OCT probe is that we can change the cannula to a minimum size of 25G (outer diameter 0.51 mm). In theory, it is possible to make a smaller probe with a suitable cannula. In fact, due to the characteristics of the structural design, our endoscopic OCT probe can be replaced with different sleeves to achieve any size from 13G (external diameter 2.41 mm) to 25G (external diameter 0.51 mm) to meet different visual field imaging requirements and different safety requirements.

In this study, a micro intraocular OCT probe was fabricated using a piezoelectric tube with quartered outside electrodes as a driver, which could realize Lissajous scanning of the movement track at the end of a single mode fiber and enter the eyeball through the scleral opening to perform real-time tomography of the fundus retina. Due to the particularity of the structural design, the outer diameter of the endoscopic OCT probe in this study can be replaced by 13G (outer diameter 2.41 mm)- 25G (outer diameter 0.51 mm), to achieve different imaging field sizes and ensure safe access to the ocular scleral incision. In this paper, the principle, theoretical derivation, and feasibility analysis of the application of a piezoelectric tube with quartered outside electrodes on the snooping probe in OCT are introduced, as well as the design characteristics, practical application and imaging situation of the endoscopic OCT probe are introduced, and the future direction of further research has prospected.

## 2 Principle derivation and feasibility analysis

### 2.1 Introduction to the principle of piezoelectric ceramics

Piezoelectric ceramics are a class of crystalline materials (Li et al., 1999). The compression or elongation caused by mechanical pressure will cause the two ends of the piezoelectric ceramics to produce different charges, resulting in voltage differences (Ravez and Simon, 2000). As a reversible, adding different voltages to the two ends of the piezoelectric ceramic produces a voltage difference between the two ends, which will also result in mechanical displacement or stress (Fatikow, 1996).

**FIGURE 1:**
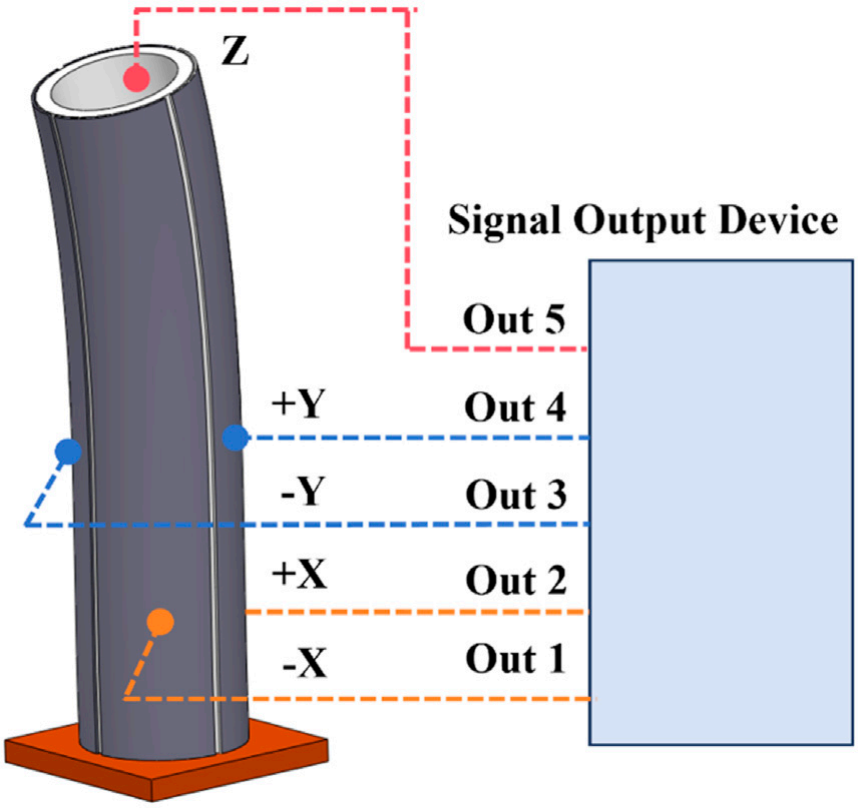
Piezoelectric tube with quartered outside electrodes.

The piezoelectric tube with quartered outside electrodes is a hollow cylindrical piezoelectric ceramic tube. A conductive coating is coated on the inner cylinder surface of the piezoelectric ceramic tube to cover the electrode inside the piezoelectric ceramic tube as a common electrode. In the outer layer, the circumference of the piezoelectric ceramic tube is evenly divided into four quadrants, each of which is coated with electrodes, the area is equal, but the electrodes are insulated from each other, and the electrodes are independent of each other. As shown in Figure 1, when the voltage signal with the same amplitude and opposite symbol is added to the -X and +X poles, the piezoelectric ceramic can be deflected in the *X* direction. If the sinusoidal voltage signal of a certain frequency is applied, the voltage ceramic tube can be repeatedly deflected by a certain frequency to achieve vibration in the *X* direction. Similarly, a voltage signal of a certain frequency with the same amplitude and opposite symbol is applied to the poles of -Y and +Y in the *Y* direction, and the voltage ceramic tube can achieve repeated deflection according to a certain frequency to achieve vibration in the *Y* direction. When two voltage signals of a certain frequency are applied in the *X* and *Y* directions at the same time, the two-dimensional vibration deflection in the *X* and *Y* directions of the piezoelectric tube is realized. To use the piezoelectric tube with quartered outside electrodes correctly and rationally, the dynamics analysis is carried out below (Qin et al., 2007).

### 2.2 Kinetic analysis and derivation of piezoelectric ceramic tube

**FIGURE 2:**
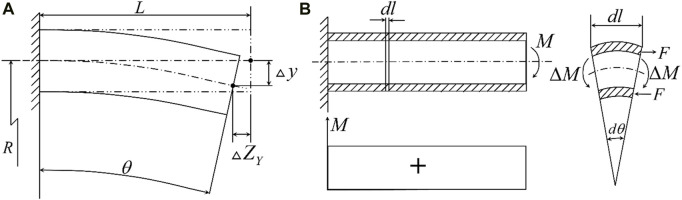
**(A)** Geometric relationship before and after *X* and *Y*-axis deformation; **(B)** Force relation after deformation of the *Y*-axis.

The following is the theoretical analysis and calculation of the deflection of the piezoelectric tube with quartered outside electrodes. As shown in Figure 2A, the extension of the *z*-axis can be expressed as Eq. 1 (Chen, 1992)

$$\Delta L=d_{31}LU/t$$ (1)

Where $d_{31}$ is the axial strain coefficient of the piezoelectric ceramic tube; L is the length of the piezoelectric ceramic tube; U is the voltage between electrodes outside the piezoelectric ceramic tube; t is the wall thickness of the ceramic pipe.

When the piezoelectric ceramics are extended axially under the action of the electric field, the wall thickness will also change, and the wall thickness change can be expressed as Eq. 2

$$\Delta t=d_{33}U$$ (2)

Where $d_{33}$ is the strain coefficient of the piezoelectric ceramic tube in the polarization direction.

The *z*-axis strain can be expressed as Eq. 3

$$\varepsilon_{z}=\Delta L/L$$ (3)

For the *X* and *Y* directions, when a voltage with the same amplitude and opposite sign is applied to the opposite two electrodes, the part applying a positive electric field will extend $\Delta S_{1}$ , while the part responsible for applying a negative electric field will shorten $\Delta S_{2}$ , which is expressed as Eq. 4

$$\Delta S_{1}=\Delta S_{2}=\Delta S$$ (4)

when the positive and negative electric fields are equal.

As shown in Figure 2A, the displacement △y in the *Y*-axis direction is represented as Eq. 5

$$△y=R\left(1-\cos\theta \right)\approx R\theta^{2}/2$$ (5)

When the *Y*-axis is moving, the coupling caused in the *Z*-axis is expressed as Eq. 6

$$△Z_{Y}=L-R\sin\theta \approx L\theta^{2}/6$$ (6)

Let $\Delta S^{0}$ be the displacement generated when the electric field in the same electric field strength is applied, which can be expressed as

$$\Delta S_{0}=d_{31}LU/t$$ (7)

As shown in Figure 2B, the differential element dl was taken for analysis, and the deformation produced by the differential element dl was ±dS . The external forces generated by the surface of the PZT tube were ±F when the PZT tube was operated under driving voltages of the same magnitude and opposite sign. This pair of forces of equal magnitude and opposite directions form a couple whose equivalent bending moment is let be ΔM . The PZT tube undergoes bending deformation under the action of this couple, and dθ is the end Angle. According to the mechanics of materials, Eqs 8, 9 is established:

$$d\theta =\frac{\Delta M}{EI}dl$$ (8)

$$\Delta M=M=F\cdot D_{0}$$ (9)

Where D is the outer diameter of the PZT tube; d is the inner diameter of the PZT tube; $D_{0}$ is the distance between the centroid of the two deformed members in the *Y*-axis direction, $D_{0}=\left(D+d\right)/2$ ;

According to the relationship between stress and strain in material mechanics, Eq. 10 can be obtained:

$$\frac{F}{A}=\sigma =\varepsilon E=\frac{\Delta S_{0}}{L}E=\frac{dS}{dl}E$$ (10)

Where E is the elastic modulus of the PZT tube; I is the Second moment of area of the PZT tube, $I=\left[\pi \left(D^{4}-d^{4}\right)\right]/64$ ; A is the area under force, $A=\left[\pi \left(D^{2}-d^{2}\right)\right]/16$ ; L is the length of the PZT tube; σ represents the stress of the material in pascals (Pa) and represents the ratio of the force applied inside the material to its unit area; ε represents the strain of the material, no unit, and represents the ratio of the change in the length of the material to the original length.

Eqs 11–13 can be obtained by static analysis:

$$d\theta =\frac{AD_{0}}{I}dS$$ (11)

$$R=\frac{dl}{d\theta }=\frac{\left(D^{2}+d^{2}\right)L}{4D_{0}\Delta S_{0}}$$ (12)

$$\theta =\frac{AD_{0}}{I}\Delta S_{0}=\frac{4D_{0}}{D^{2}+d^{2}}\Delta S_{0}$$ (13)

where $D_{0}$ is the distance between the centroids of the two deformed members in the *Y*-axis; D is the outer diameter of the piezoelectric ceramic tube; d is the inner diameter of the piezoelectric ceramic tube; R is the bending radius of the ceramic tube; θ is shown in Figure 2A.

The end of the piezoelectric ceramic tube should be connected to a single-mode fiber cantilever beam. If the length of the single-mode fiber cantilever beam is $l_{t}$ , the transverse displacement is:

$$\Delta y=\frac{2D_{0}L\Delta S_{0}}{D^{2}+d^{2}}=\frac{\left(D+d\right)d_{31}L^{2}U}{D^{2}+d^{2}}$$ (14)

$$\Delta y^{\prime }=\Delta y+l_{t}\theta$$ (15)

Bring Formulas 12–14 into (Eq. 15) to get

$$\Delta y^{\prime }=\frac{2D_{0}L+4D_{0}l_{t}}{D^{2}+d^{2}}\Delta S_{0}$$ (16)

In theory, Δx and Δy are not coupled, so it can be considered that they have the same displacement equation. By substituting Eq. 7 into Eq. 16, displacement equations in *X* and *Y* directions can be obtained. The displacement equations of the end of the piezoelectric ceramic tube in three directions Eqs 17–19 are obtained by considering the coupling caused by the lateral displacement in the *Z* direction.

$$\Delta x^{\prime }=\frac{2D_{0}L+4D_{0}l_{t}}{D^{2}+d^{2}}\cdot d_{31}U_{x}/t$$ (17)

$$\Delta y^{\prime }=\frac{2D_{0}L+4D_{0}l_{t}}{D^{2}+d^{2}}\cdot d_{31}U_{y}/t$$ (18)

$$\Delta z^{\prime }\approx \Delta z=d_{31}U_{Z}L/t$$ (19)

Eq. 19 is approximately the displacement in the *Z*-axis direction of the PZT tube without deflection, which can be approximately regarded as $\Delta z^{\prime }$ is proportional to $U_{Z}$ .

Through the dynamic analysis of the whole, when the PZT tube drives the end of the single-mode fiber to scan, the certain displacement of the end of the single-mode fiber in the x, y, and z directions is proportional to the $\Delta x^{\prime }$ , $\Delta y^{\prime }$ , $\Delta z^{\prime }$ , respectively. Therefore, the vibration of the piezoelectric tube with quartered outside electrodes and the end of the single-mode fiber can be improved by adjusting the signal voltage.

### 2.3 Analysis and derivation of vibration model of single-mode fiber cantilever beam

**FIGURE 3:**
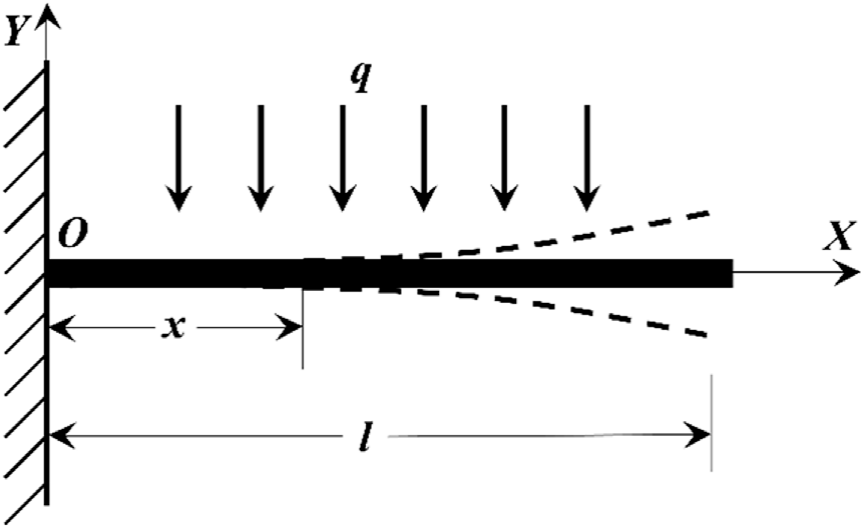
Cantilever beam structure diagram.

A cantilever beam is a structure with one end fixed and another suspended. Compared with the fixed end that does not generate axial or vertical displacement, the suspended free end can generate both axial and vertical forces. The simplified model is shown in Figure 3. The fiber cantilever beam is a fixed fiber end, and the other end of the suspended fiber makes it a free end. In the case that the single-mode fiber is not affected by gravity and the external environment, the fiber cantilever is in a horizontal state (that is, reconnected with the *X*-axis). When there is no vibration in the external environment, the fiber is only affected by gravity, currently, the external load is 0, and the load of the cantilever beam is only the gravity of the cantilever beam itself, currently, the cantilever beam is in a free state. As shown in Figure 3, the coordinate system shown above is established. The *X*-axis overlaps with the cantilever beam when the cantilever beam is stationary and not affected by gravity. O is the origin of the coordinate system, the total length of the cantilever beam is l, the distance from any point of the cantilever beam to the fixed end is x, the uniform load is q, and the arbitrary angle of the fiber optic cantilever beam is θ.

The mechanical model of the Euler-Bernoulli beam is used in the theoretical analysis of the natural frequency of a fiber optic cantilever beam. The vibration of the cantilever beam belongs to the vibration of the continuous elastomer in material mechanics, so the vibration of the cantilever beam has infinite degrees of freedom and corresponding natural frequency and principal mode. The final vibration of the cantilever beam can be expressed as the superposition of infinite principal mode. When the cantilever beam is forced to vibrate due to the external environment, only the deformation caused by bending is considered, and the influence of the deformation caused by shear and the moment of inertia is ignored, which conforms to the mechanical model analysis of Euler-Bernoulli beam (Necla and Süleyman, 2016).

As can be seen from the structural diagram of the cantilever beam in Figure 3, one end of the cantilever beam is a fixed end, and the other end is free. The single-mode fiber is recombined with the *X*-axis without gravity, and the differential equation of the cantilever beam motion (Eq. 20) (Liu, 2003; Ohm et al., 2006; Sun et al., 2009) is:

$$EI\frac{\partial^{4}w\left(x,t\right)}{\partial x^{4}}+\rho A\frac{\partial^{2}w\left(x,t\right)}{\partial t^{2}}=0$$ (20)

Where E is the elastic modulus of single-mode fiber, it can be seen from the data that $E=7.4668\times 10^{10}Pa$ ; I is the Second moment of area of the optical fiber, $I=\left(\pi D^{4}\right)/64$ ; D is the diameter of the optical fiber 125 μm; A is the optical fiber cross-section area; ρ is the density of conventional communication single-mode fiber, $\rho =2203kg/m^{3}$ ; x is the distance of a beam section from the origin O point; $w\left(x,t\right)$ is the displacement of the beam section at time t at the distance x from the origin O point; t there stands for a moment in time.

The differential equation has the fourth partial derivative with respect to x and the second partial derivative with respect to t , so it requires four boundary conditions and two initial conditions to solve. The boundary conditions of the cantilever beam (Fang and Zhang, 2011; Yang et al., 2011) are denoted as Eqs 21–24.

$$w\left(x=0\right)=0$$ (21)

$${\left.\frac{dw}{dx}\right|}_{x=0}=0$$ (22)

$${\left.\frac{\partial^{2}w}{\partial x^{2}}\right|}_{x=l}=0$$ (23)

$$\frac{\partial }{\partial x}\left(\text{EI}\frac{\partial^{2}w}{\partial x^{2}}\right){|}_{x=l}=0$$ (24)

The vibration mode of the system is independent of time, so the equation can be solved by the method of separating variables, and the solution of the equation can be separated by variables, and the free vibration solution of the partial differential equation can be obtained as Eq. 25:

$$w\left(x,t\right)=W\left(x\right)T\left(t\right)$$ (25)

By substituting this solution into the differential equation of motion of a cantilever beam, Eq. 26 is obtained:

$$\left\{\begin{array}{l} W\left(x\right)=C_{1}\cos\beta x+C_{2}\sin\beta x+C_{3}\cosh\beta x+C_{4}\sinh\beta x\\ T\left(t\right)=A\cos wt+B\sin wt \end{array}\right.$$ (26)

where $\beta^{4}=\frac{\rho Aw^{2}}{\text{EI}}$ .

Bring the boundary condition Eqs 21, 22 into the above formula to get Eq. 27:

$$C_{1}+C_{3}=0,C_{2}+C_{4}=0$$ (27)

Further sorting can obtain Eq. 28:

$$W\left(x\right)=C_{1}\left(\cos\beta x-\cosh\beta x\right)+C_{2}\left(\sin\beta x-\sinh\beta x\right)$$ (28)

Then the boundary conditions Eqs 23, 24 are brought into the equation:

$$\left\{\begin{array}{l} -C_{1}\left(\cos\beta l+\cosh\beta l\right)-C_{2}\left(\sin\beta l+\sinh\beta l\right)=0\\ -C_{1}\left(-\sin\beta l+\sinh\beta l\right)-C_{2}\left(\cos\beta l+\cosh\beta l\right)=0 \end{array}\right.$$ (29)

So, the resulting frequency equation is Eq. 30:

$$\cos\left(\beta_{n}l\right)\cosh\left(\beta_{n}l\right)=-1$$ (30)

the root $\beta_{n}l$ of this equation represents the natural frequency of the vibration system.

$$w_{n}={\left(\beta_{n}l\right)}^{2}{\left(\frac{\text{EI}}{\rho Al^{4}}\right)}^{\frac{1}{2}},n=1,2,3\ldots$$ (31)

The value of $\beta_{n}l\left(n=1,2,3\ldots \right)$ satisfying the above Eq. 31 is Eq. 32:

$$\begin{array}{l} \beta_{1}l=1.875104,\beta_{2}l=4.694091,\beta_{3}l=7.854757,\\ \beta_{4}l=10.995541,\beta_{5}l=14.1372 \end{array}$$ (32)

If the value of $C_{2}$ relative to $\beta_{n}$ is expressed as $C_{2n}$ , according to $C_{1n}$ in the formula, $C_{2n}$ can be expressed as Eq. 33:

$$C_{2n}=-C_{1n}\left(\frac{\cos\beta_{n}l+\cosh\beta_{n}l}{\sin\beta_{n}l+\sinh\beta_{n}l}\right)$$ (33)

The principal mode function corresponding to the natural frequency of order n (Eq. 34) can be obtained:

$$\begin{array}{l} W_{n}\left(x\right)=C_{1n}\left[\left(\cos\beta_{n}l-\cosh\beta_{n}l\right)-\frac{\cos\beta_{n}l+\cosh\beta_{n}l}{\sin\beta_{n}l+\sinh\beta_{n}l}\left(\sin\beta_{n}x-\sinh\beta_{n}x\right)]\right.\\ n=1,2,3... \end{array}$$ (34)

Thus, the first five natural frequencies of the cantilever beam (Eq. 35) can be obtained, and n = 1,2,3,4,5 can be brought into the equation:

$$\begin{array}{l} \omega_{1}=1.875104^{2}{\left(\frac{\text{EI}}{\rho Al^{4}}\right)}^{\frac{1}{2}},\omega_{2}=4.694091^{2}{\left(\frac{\text{EI}}{\rho Al^{4}}\right)}^{\frac{1}{2}},\omega_{3}=7.854757^{2}{\left(\frac{\text{EI}}{\rho Al^{4}}\right)}^{\frac{1}{2}}\\ \omega_{4}=10.995541^{2}{\left(\frac{\text{EI}}{\rho Al^{4}}\right)}^{\frac{1}{2}},\omega_{5}=14.1372^{2}{\left(\frac{\text{EI}}{\rho Al^{4}}\right)}^{\frac{1}{2}} \end{array}$$ (35)

Where w=2πf , f is the natural frequency of vibration of the cantilever beam.

**FIGURE 4:**
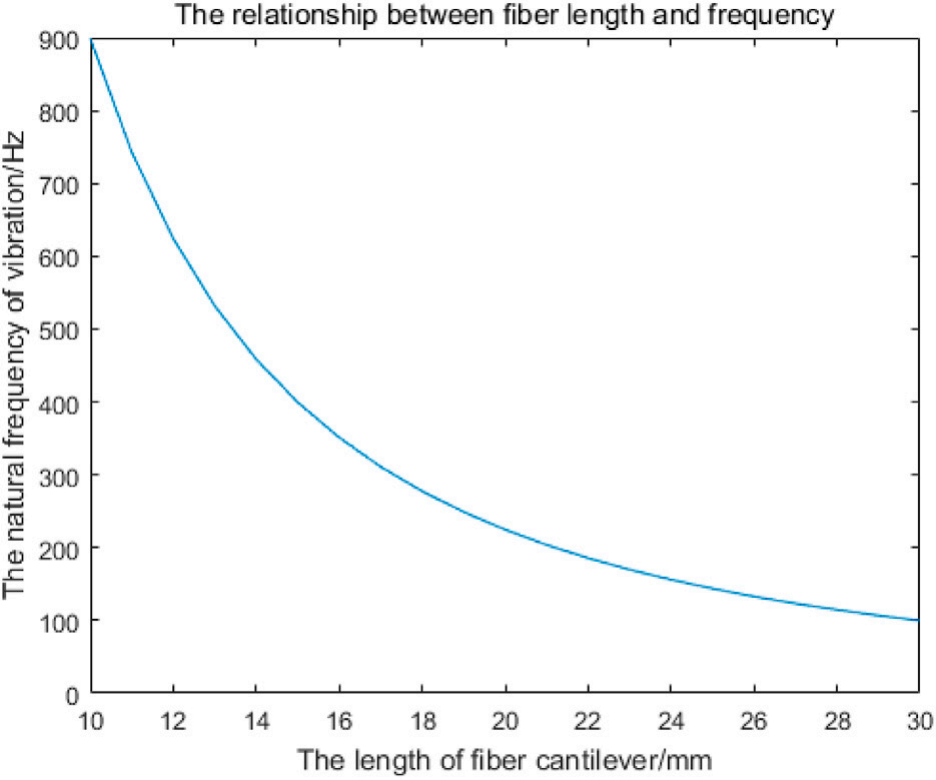
Relationship between the length of the single-mode fiber cantilever beam and the first-order natural frequency.

As shown in Figure 4, the natural frequencies of fiber cantilever beams with different lengths can be obtained by MATLAB simulation. Since the resonant frequency of the cantilever beam is mainly determined by the first-order natural vibration frequency, the first-order vibration frequency of the cantilever beam with different lengths is shown in Table 1.

**TABLE 1:** First-order vibration frequencies of single-mode fiber cantilever beams of different lengths.

Fiber optic cantilever length/unit mm	Resonance frequency/unit Hz
10	898.8
11	742.8
12	624.4
13	531.8
14	458.6
15	399.5
16	351.1
17	311.0
18	277.4
19	249.0
20	224.7
21	203.8
22	185.7
23	169.9
24	156.0
25	143.8
26	133.0
27	123.3
28	114.6
29	106.9
30	99.9

The single-mode fiber cantilever beam is regarded as a stressed object with a uniform load. As shown in Figure 3, the fiber is rejoined with the *X*-axis without gravity. In the range of 0≤x≤l , the bending moment equation of the single-mode fiber cantilever beam is Eq. 36:

$$M\left(x\right)=-\frac{1}{2}q{\left(l-x\right)}^{2}$$ (36)

Where $M\left(x\right)$ is the natural offset of the fiber cantilever of different lengths.

The differential equation and Angle equation of the deflection curve Eqs 37–39 are as follows:

$$\frac{d^{2}y}{dx^{2}}=\frac{M\left(x\right)}{\text{EI}}$$ (37)

$${\text{EIy}}^{\prime \prime }=-\frac{1}{2}qx^{2}+qlx-\frac{1}{2}ql^{2}$$ (38)

$$\text{EI}\theta =-\frac{1}{6}qx^{3}+\frac{1}{2}qlx^{2}-\frac{1}{2}ql^{2}x+C$$ (39)

Integrate Formula 39 to obtain:

$$\text{EIy}=-\frac{1}{24}qx^{4}+\frac{1}{6}qlx^{3}-\frac{1}{4}ql^{2}x^{2}+\text{Cx}+D$$ (40)

It can be seen from the boundary conditions that when x=0 , y=0 , θ=0 . Therefore, the integral constants C = 0 and D = 0 in (Eq. 40) can be determined, that is, the Angle and deflection of the fiber cantilever beam at the fixed point are both 0. Eq. 40 can be simplified as Eq. 41:

$$\text{EI}y=-\frac{1}{24}qx^{4}+\frac{1}{6}qlx^{3}-\frac{1}{4}ql^{2}x^{2}$$ (41)

The equation that can finally obtain the offset is Eq. 42:

$$y=-\frac{qx^{2}}{24\text{EI}}\left(x^{2}-4lx+6l^{2}\right)$$ (42)

When x=l , the offset y (Wang et al., 2013; Zhang et al., 2013; Xu et al., 2014) has a maximum value, which is Eq. 43:

$$y_{\max}=-\frac{ql^{4}}{8\text{EI}}$$ (43)

When the external force is not applied to the cantilever beam, the load is only the gravity of the fiber cantilever beam itself. q is the uniform load, that is, the gravity per meter of the cantilever beam, so the above equation can be rewritten as (Beléndez et al., 2008):

$$y_{\max}=-\frac{Wl^{3}}{8\text{EI}}$$ (44)

Where W is the gravity of the cantilever beam itself, W=ql .

$$W=\rho Vg=\rho \frac{\pi D^{2}}{4}\mathrm{lg}$$ (45)

Bring the Formula 45 into (Eq. 44) to simplify:

$$y_{\max}=-\frac{2\rho gl^{4}}{{\text{ED}}^{2}}$$ (46)

Where $y_{\max}$ is the maximum offset of the cantilever beam; g is the acceleration of gravity, $g=9.8m/s^{2}$ ; l is the length of the fiber optic cantilever; I is the Second moment of area, $I=\left(\pi D^{4}\right)/64$ .

The minus sign in Eq. 46 indicates only the direction. It can be seen from Eq. 46 that as the length of the optical fiber cantilever increases, the offset of the optical fiber cantilever increases. However, when the length of the fiber cantilever beam is very small, the vibration amplitude of the fiber cantilever beam is too small when measuring the vibration signal, the light intensity received by the receiving end will hardly change, and the imaging effect will be greatly reduced, so it is necessary to find the appropriate length of the fiber cantilever beam at the transmitting end. Considering all aspects, the length of single-mode fiber cantilever beam is 22–23 mm.

## 3 Study and design of endoscopic OCT probe

### 3.1 Mechanical structure design of endoscope OCT probe

The resonance frequency and the maximum amplitude of the optical fiber cantilever beam are analyzed theoretically. The probe was designed to fix the treated single-mode optical fiber in the center position of the piezoelectric tube with quartered outside electrodes by designing 3D printed units and fixed with glue using precision instruments.

To reduce the cost of the piezoelectric tube with quartered outside electrodes, the probe structure was innovatively optimized. The probe no longer installs the piezoelectric ceramic tube in the probe tube wall, thus removing the limitation of the probe size on the piezoelectric tube with quartered outside electrodes, and there is no need to customize the piezoelectric tube with quartered outside electrodes of specific size, thus greatly reducing the manufacturing cost of the probe. Through the theoretical simulation analysis of the single-mode fiber cantilever beam, the fiber extension length is set at about 22–23 mm, so that the extended single-mode fiber is completely under the protection of the medical stainless-steel probe.

**FIGURE 5:**
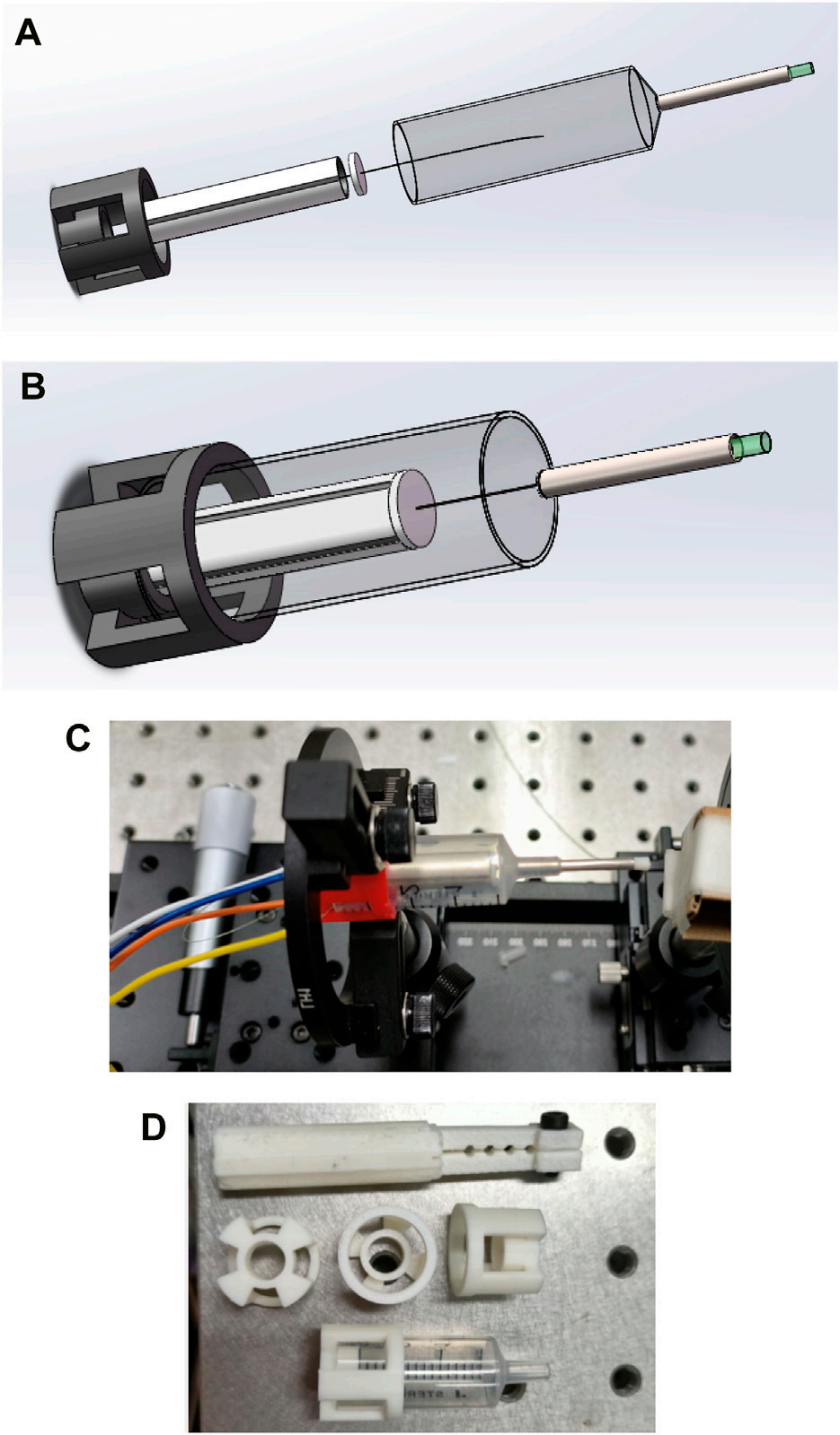
**(A)** Structure diagram of the endoscope OCT probe designed in this study; **(B)** A schematic depiction of the completed encapsulation process for the endoscopic OCT probe designed in this study; **(C)** Mechanical structure drawing of the physical object; **(D)** Some units of the endoscope OCT probe are 3D printed.

Through Gaussian beam modeling and simulation, the GRIN lens with appropriate parameters is selected to make the divergent beam emitted from the end of the single-mode fiber get a good focus, and at the same time, the optimal distance between the end of the single-mode fiber and the GRIN lens and the optimal imaging distance from the GRIN lens to the imaging object is determined, so as to effectively improve the imaging quality. The GRIN lens is fixed at the front end of the medical stainless-steel probe tube, and the GRIN lens maintains the simulated optimal distance from the end of the single-mode fiber, so that the scanning beam of the single-mode fiber is focused through the GRIN lens and resonates with the single-mode fiber at a certain frequency and a certain trajectory to achieve high-quality imaging, as shown in Figure 5.

### 3.2 COMSOL modeling was performed for resonance analysis

The equations should be inserted in editable format from the equation editor.

After the preliminary mechanical structure design of the endoscope OCT probe was completed, the modeling analysis was performed using COMSOL, considering that the actual situation was different from the theoretical analysis of the single-mode fiber cantilever beam analyzed previously. Because the single-mode fiber is processed using precision instruments, it is fixed with glue at the center of the piezoelectric tube with quartered outside electrodes, rather than a bare fiber.

The piezoelectric tube with quartered outside electrodes, single-mode optical fibers, some 3D printed units, and adhesions for precision instruments (used in connection sites) were modeled using COMSOL. The density, Young’s modulus, Poisson’s ratio, and other material parameters of each part were respectively input to conduct full-true modeling. The characteristic frequency analysis of the built model was carried out to obtain the resonance frequency at this time.

**FIGURE 6:**
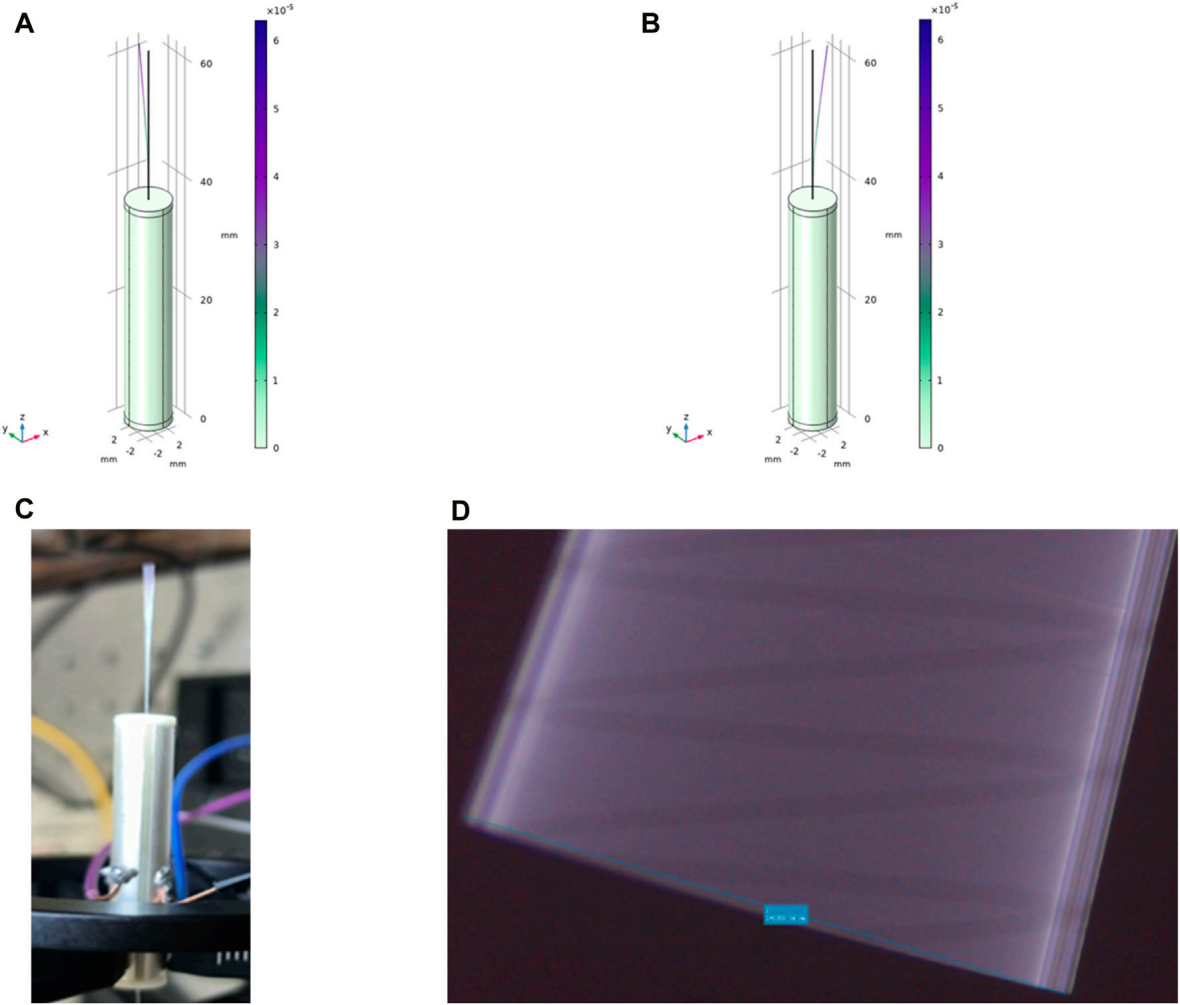
**(A)** In COMSOL modeling, the resonant frequency is 162.91 Hz, and the vibration amplitude of 2–3 mm appears at the end in the y-z plane; **(B)** The resonant frequency in COMSOL modeling is 162.9 Hz, and the vibration amplitude of 2–3 mm appears at the end in the x-z plane; **(C)** The vibration of the end of the single-mode fiber is clearly and directly observed with the bare eye; **(D)** The maximum vibration amplitude of the single-mode fiber can reach 2360 μm after obvious observation and measurement under the electron microscope.

As shown in Figure 6A, when the frequency is 162.91 Hz and the length of the single-mode fiber cantilever beam is 23.5 mm, COMSOL modeling analysis shows that the fiber end has an obvious vibration amplitude of 2–3 mm in the y-z plane. As shown in Figure 6B, when the frequency is 162.9 Hz and the length of the single-mode fiber cantilever beam is 23.5 mm, the end of the fiber appears obvious vibration in the x-z plane, and the vibration amplitude is 2–3 mm, which meets the predetermined requirements. As shown in Figure 6C, after applying the voltage signal, the obvious vibration at the end of the single-mode fiber can be directly observed. As shown in Figure 6D, the maximum vibration amplitude of single-mode fiber can reach 2360 μm after accurate observation and measurement under the electron microscope by applying the above voltage signal.

### 3.3 Study on Lissajous scanning trajectory at the end of single-mode fiber

**FIGURE 7:**
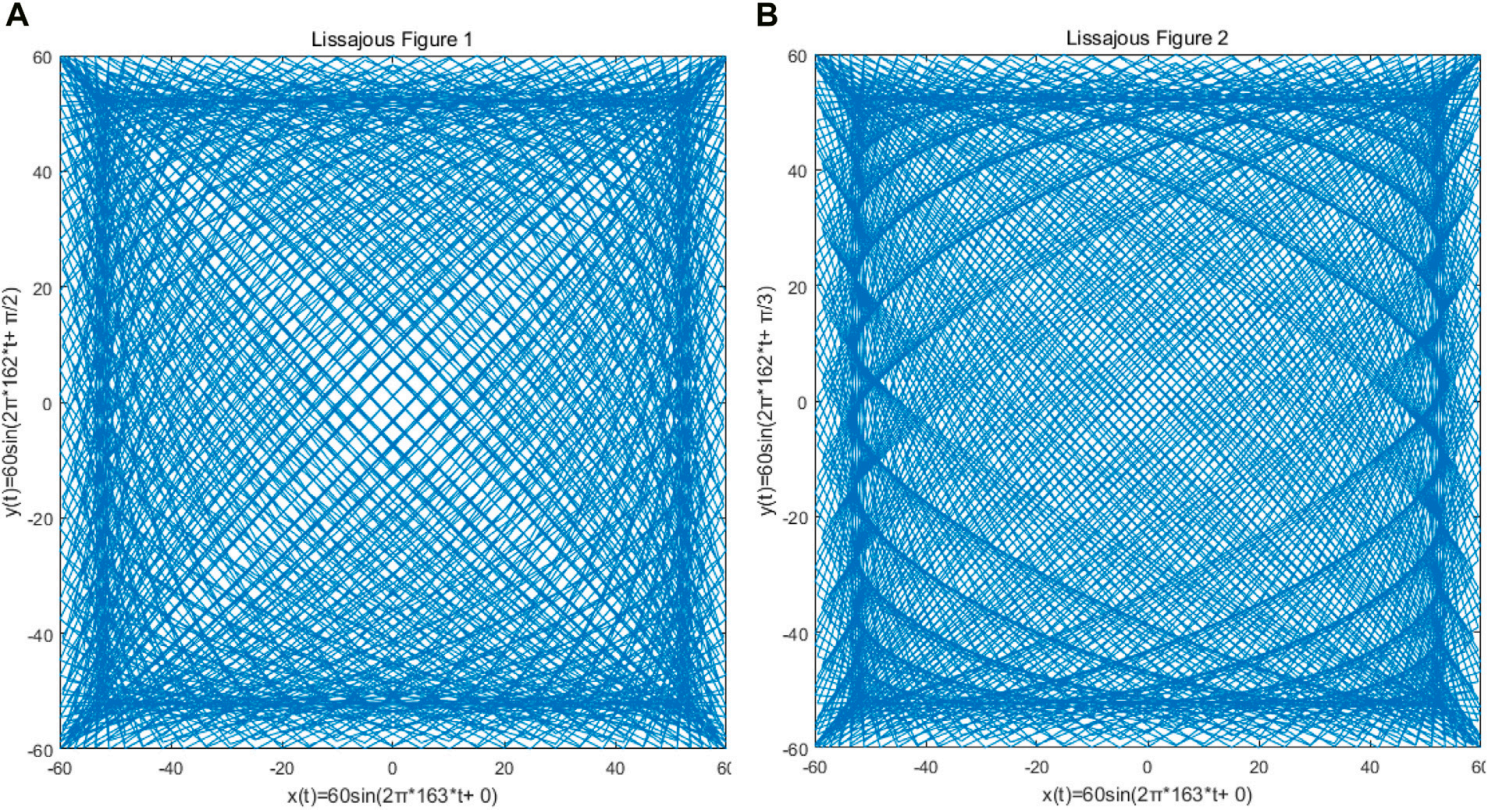
Because of the different phase difference of *x* and *y* direction signals of the Lissajous locus, the density of the Lissajous locus and the number of lobes are obviously different. **(A)** The phase difference between x and y signals is π/2; **(B)** the phase difference between the x and y signals is π/3.

The Lissajous trajectory was selected for the single-mode optical fiber end scanning trajectory of the OCT probe, which is driven by two orthogonal harmonic vibrations, and determined by the amplitude, frequency, and initial phase of the two harmonic vibrations. The resonant frequency of 162.9 Hz obtained by the previous resonance simulation using COMSOL is taken as a reference, and the simple harmonic vibration frequencies in the *x* and *y* directions are set to 163 Hz and 162 Hz respectively. Considering that the output voltage of the signal will be amplified to 60 V in the actual situation, the amplitudes of the two simple harmonic vibrations are set to 60 V. As shown in Figure 7, the input signal in the *x* direction will be simulated by MATLAB for scanning trajectory, and the adjustment of parameters such as density, filling rate, and lobe number of Lissajous trajectory can be realized by phase adjustment, to achieve the optimal single-mode optical fiber end scanning.

**FIGURE 8:**
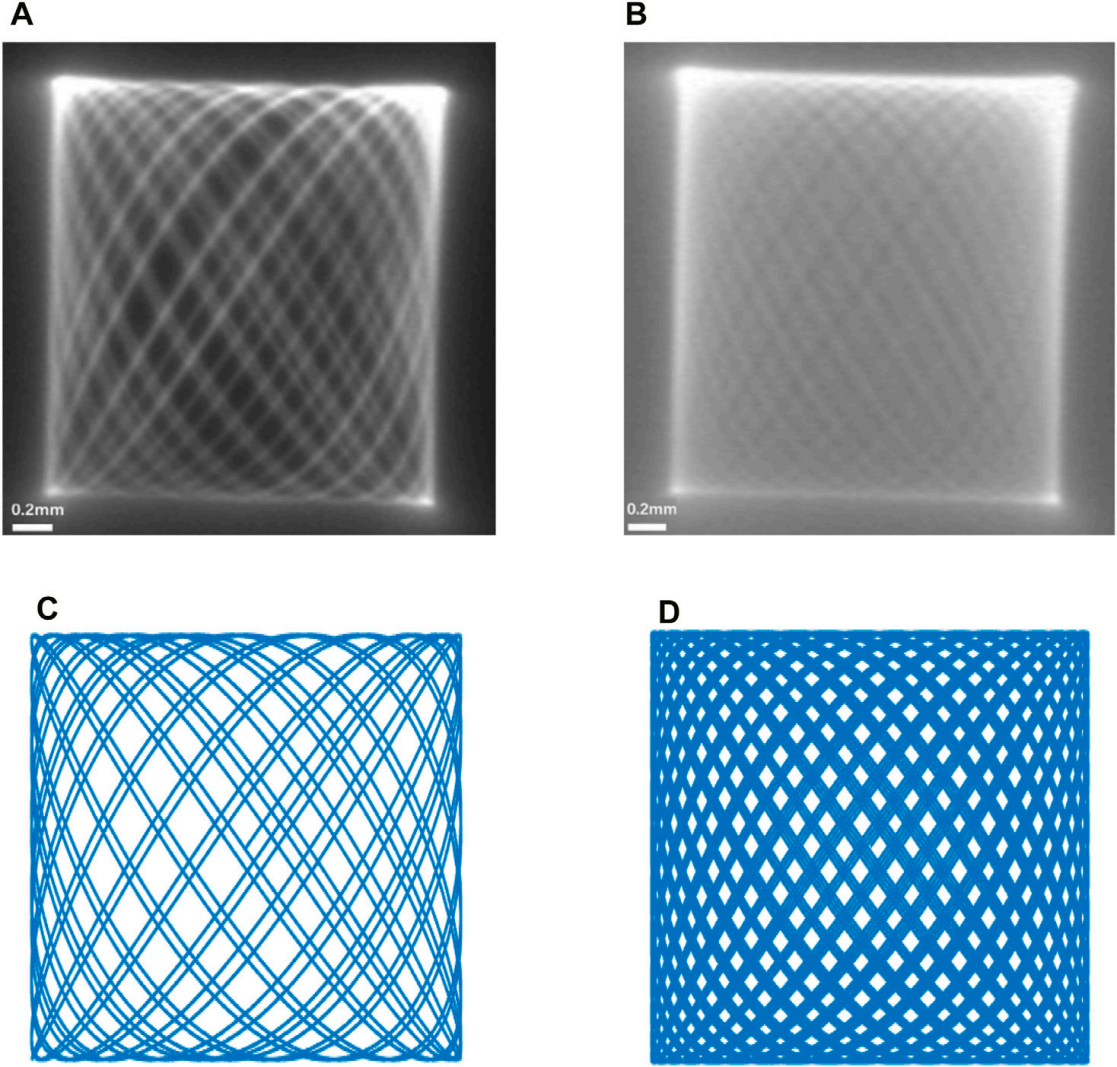
**(A,B)** are the movement trajectories of the end of the single-mode fiber taken with the COMS camera, and **(C,D)** are the simulation plots of the Lissajous trajectory carried out in MATLAB. It can be found that **(A)** achieves good contrast with **(C,B)** with **(D)**, respectively.

Considering the actual scanning situation of the endoscopic OCT probe, the end of the single-mode fiber is a focused spot with a certain actual size. By connecting the single-mode fiber to the laser and using the COMS camera for observation, the trajectory of the focused spot at the end of the fiber can be observed. As shown in Figure 8, we show the contrast between the end of a single-mode fiber with a focused spot of a certain size captured by a real CMOS camera and the simulation in MATLAB. The scanning range of the end of the single-mode fiber was 2.1 mm × 2.1 mm at this time of actual measurement.

### 3.4 Design and construction of optical path for endoscopic OCT probe

**FIGURE 9:**
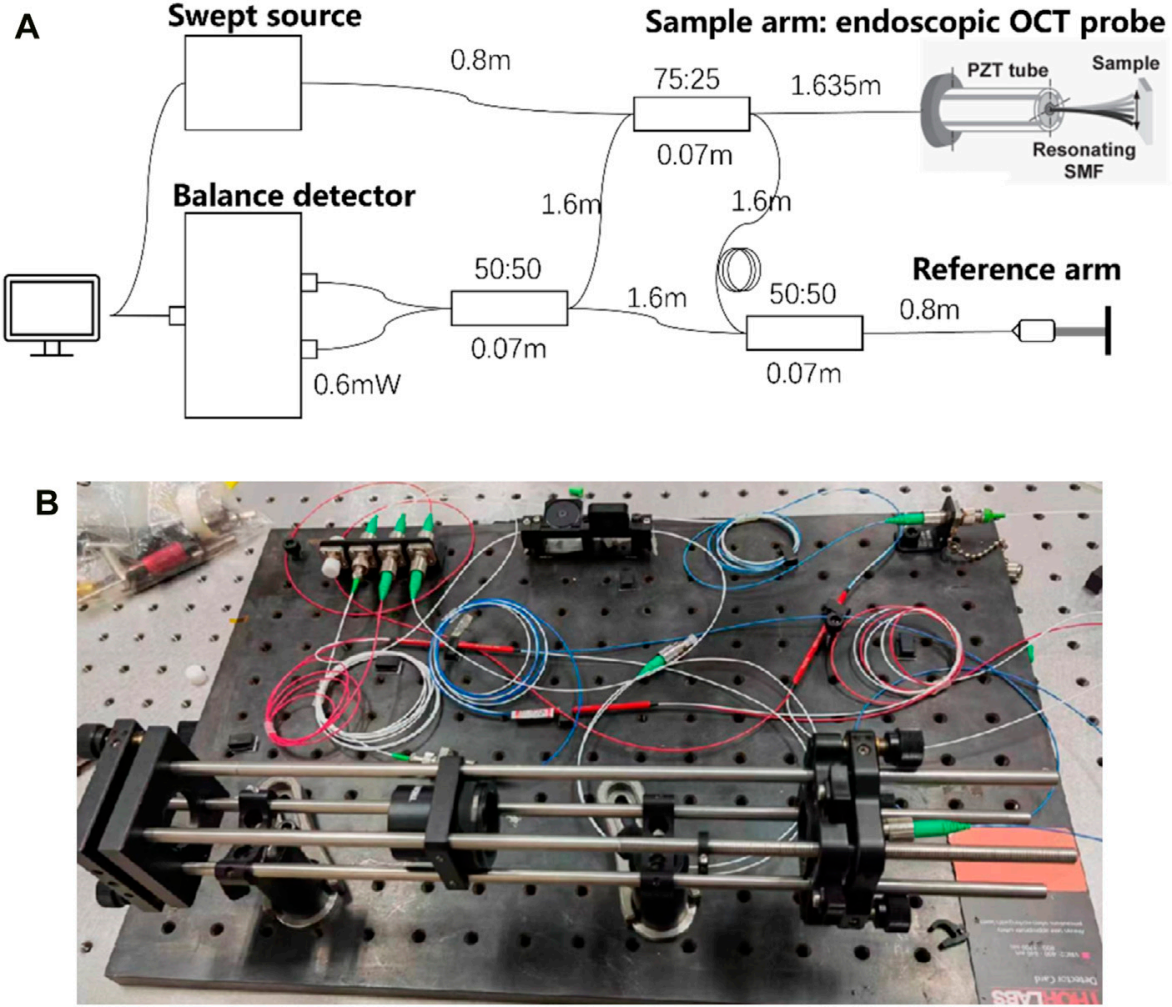
**(A)** Design of the optical path structure diagram. **(B)** The actual optical path structure.

The imaging principle of endoscopic OCT probe technology is Optical Coherence Tomography (OCT), which is based on the principle of low coherence interference of light. A reference arm is set as the reference object of vertical and horizontal depth, and the sample arm is moved for scanning. The backward-facing interference light returned from the sample at different depths is processed, and the fault structure of the tissue to be measured is obtained after processing, to achieve A-scanning, that is, a series of one-dimensional longitudinal depth information is obtained (Danielson and Boisrobert, 1991). A series of A-scans are obtained by controlling the sample arm scanning on the transverse plane, and two-dimensional images are obtained through processing and analysis, that is, B-scanning is realized. Also, a series of B-scans obtained by scanning were processed and analyzed to obtain three-dimensional images of the tested samples (Song et al., 2021). As shown in Figure 9, the optical path was designed and built, and the length of each fiber was accurately calculated to ensure the optical path matching to ensure normal imaging.

## 4 Encapsulation technology and imaging experiment of endoscopic OCT probe

### 4.1 Encapsulation technology experiment of endoscopic OCT probe

**FIGURE 10:**
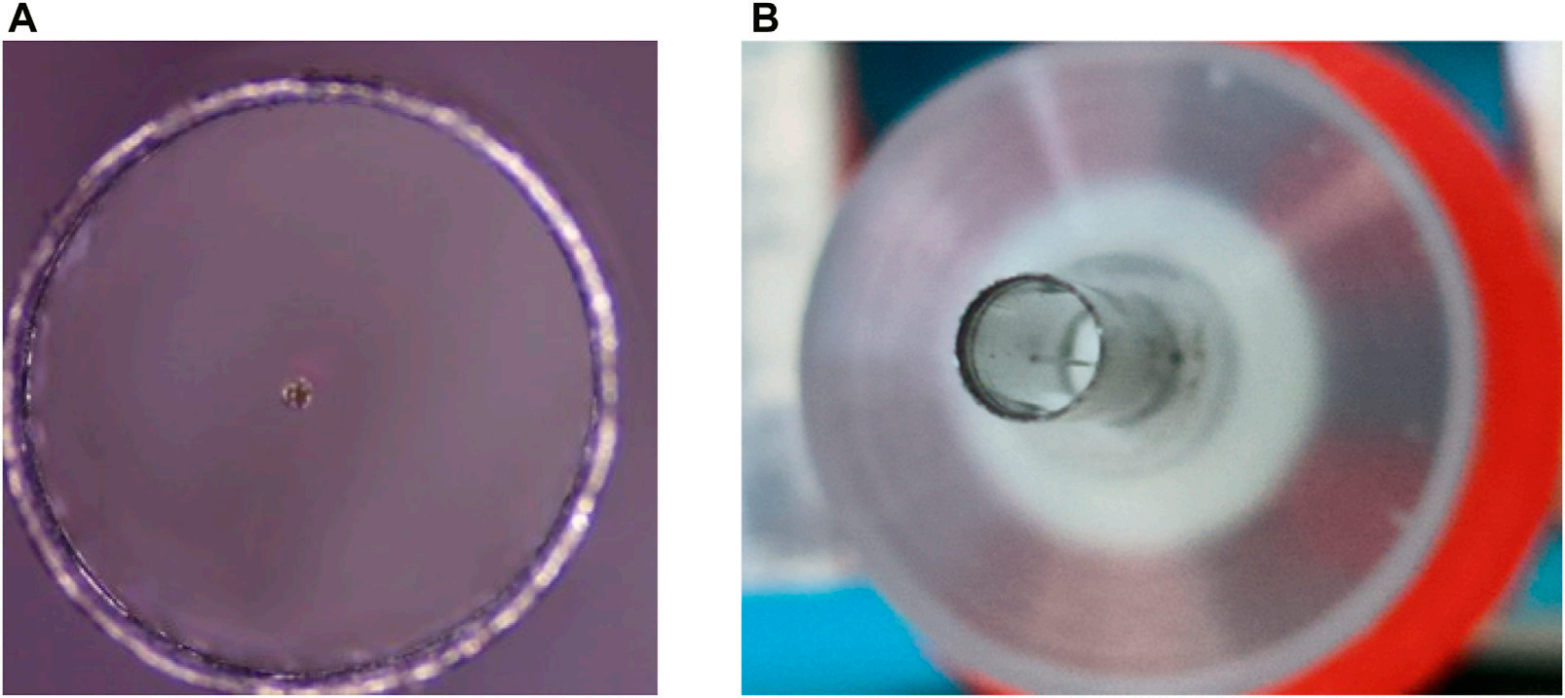
**(A)** and **(B)** are single-mode optical fibers located in the geometric center of the medical stainless-steel probe to ensure good imaging quality.

For the encapsulation of the endoscopic OCT probe, it is necessary to ensure the accuracy of the encapsulation process. As shown in Figure 10, it is ensured that the scanning optical fiber is in the geometric center of the medical stainless-steel probe, so that the resonant vibration at the end of the fiber is not affected by the medical stainless-steel probe. It is necessary to design some assembly and encapsulation devices to assist the encapsulation work. At the same time, the GRIN lens is installed for focusing, and the distance between the GRIN lens and the end of the single-mode fiber is required to ensure that the best effect of Gaussian light speed simulation is achieved. This adjustment of accuracy requires the installation of a precision displacement platform and the use of an optical power meter to find the strongest optical power to ensure the best imaging quality.

**FIGURE 11:**
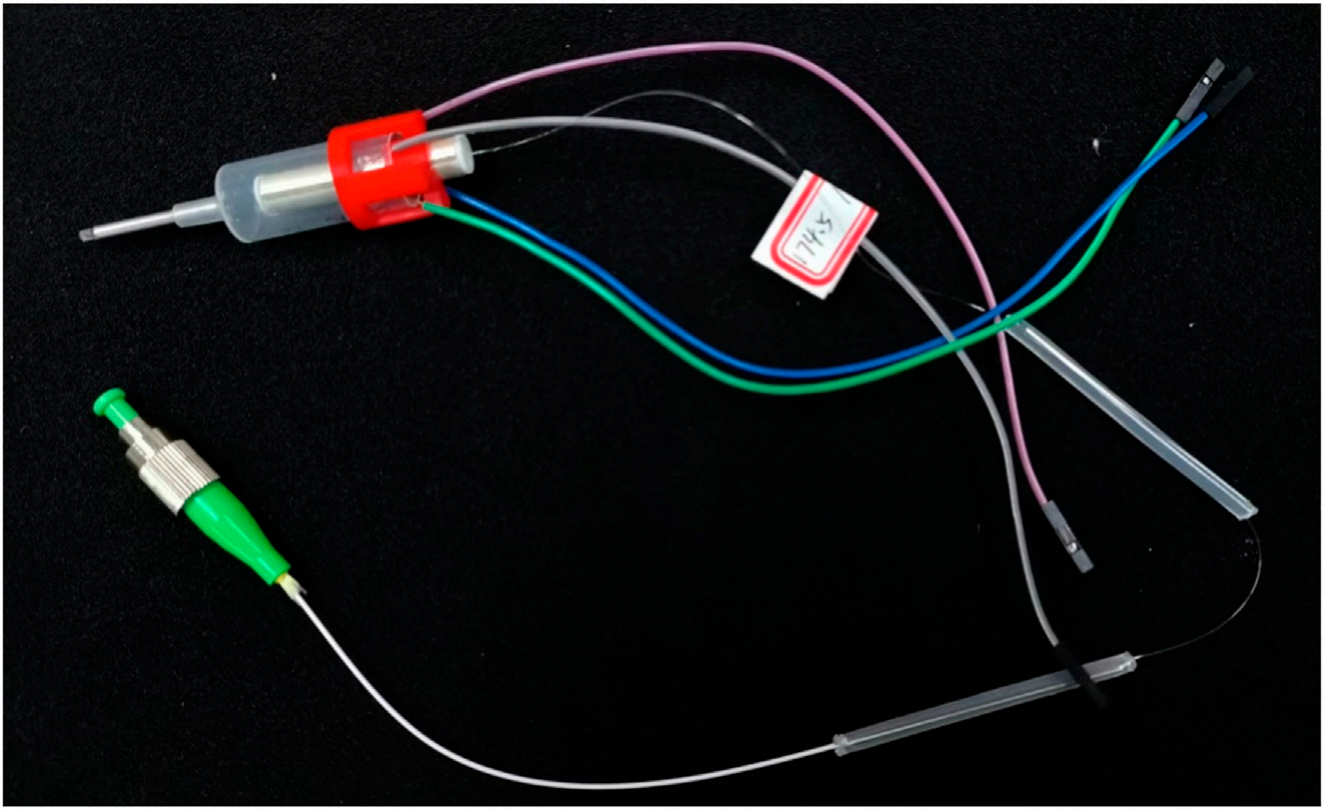
The encapsulated endoscope OCT probe can be connected to the OCT optical path and apply input signals for OCT imaging.

The preliminary encapsulation of the endoscopic OCT probe was obtained, and the finished product was shown in Figure 11.

### 4.2 Imaging experiment of endoscopic OCT probe

**FIGURE 12:**
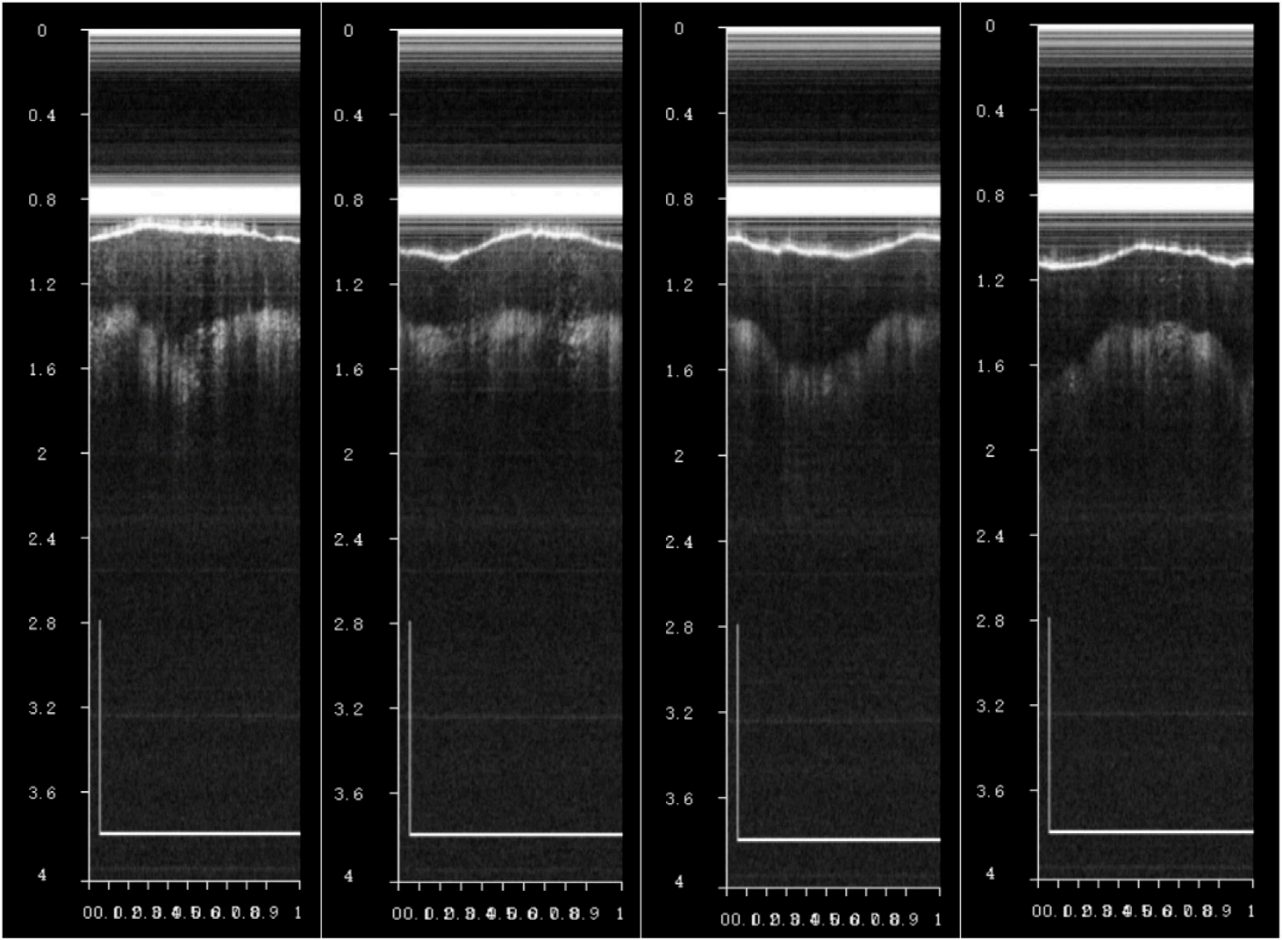
When observing a finger *in vivo* using an endoscopic OCT probe, OCT tomographic images with a distinct finger tissue stratification effect can be observed, where the scale bar is 1 mm.

The encapsulated endoscopic OCT probe was first used in the finger for imaging experiments. As shown in Figure 12, OCT tomographic images with good effect and obvious stratification of finger tissue can be obtained. The endoscopic OCT probe has not entered the eyeball *in vivo* for fundus retinal experiments in this study, which will be carried out in the future.

**FIGURE 13:**
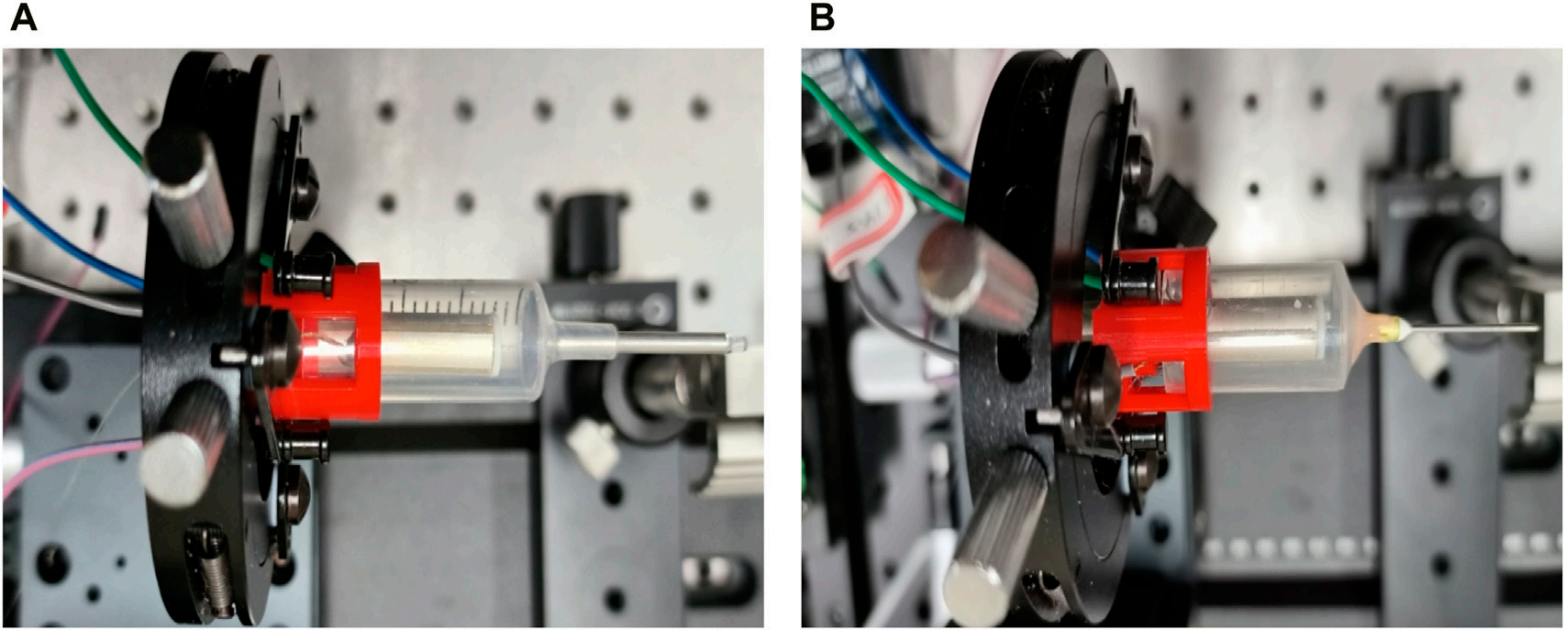
**(A)** 13G (outer diameter 2.41 mm) endoscopic OCT probe made in this study; **(B)** 25G (outer diameter 0.51 mm) endoscopic OCT probe after probe replacement.

The endoscopic OCT probe in this study can be used to adjust the vibration amplitude of the end of the single-mode fiber by controlling the applied signal voltage, to adapt to different types of 13G (outer diameter 2.41 mm)-25G (outer diameter 0.51 mm) endoscopic probes, which can be seen in Figure 13. The OCT probe size can be changed from 13G to 25G according to the work needs, which will bring different imaging field sizes. It is worth noting that GRIN lenses need to be customized to match the probe size to achieve normal beam focusing, and the encapsulation of endoscope OCT probes needs to ensure a reasonable optical distance.

## 5 Prospects for the subsequent development of endoscopic OCT probes

It is worth looking forward to the possibility that the endoscopic OCT probe can be assembled with the RCM robotic arm for subsequent automated imaging. It can be considered to realize two robotic arms for *ex vivo* ophthalmic surgery. One robotic arm is equipped with a scalpel or injection needle, and the other robotic arm is equipped with the endoscopic OCT probes from this study so that the two robotic arms can be matched for work, where the position, angle, and other parameters of surgical instruments can be displayed in real-time. This can greatly improve the safety and reliability of the robotic arm in ophthalmic surgery.

How to achieve more stable OCT scanning, how to improve the performance parameters such as the resolution of endoscopic OCT, and how to ensure the high-precision cooperation between the endoscopic OCT probe and the surgical robot will become the technical focus and difficulties in the follow-up research.

## Data Availability

The original contributions presented in the study are included in the article/supplementary material, further inquiries can be directed to the corresponding author.
